# A Neuroelectronic Interface with Microstructured Substrates for Spiral Ganglion Neurons Cultured In Vitro: Proof of Concept

**DOI:** 10.3390/bios15040224

**Published:** 2025-04-01

**Authors:** Boris Delipetar, Jelena Žarković Krolo, Ana Bedalov, Damir Kovačić

**Affiliations:** 1 Department of Physics, Faculty of Science, University of Split, Ruđera Boškovića 33, 21000 Split, Croatia; boris.delipetar.00@fesb.hr (B.D.);; 2 The Center of Research Excellence for Science and Technology Integrating Mediterranean Region (STIM), University of Split, Ruđera Boškovića 31, 21000 Split, Croatia; 3 The Doctoral Program in Mechanical Engineering, Faculty of Electrical Engineering, Mechanical Engineering and Naval Architecture, University of Split, Ruđera Boškovića 32, 21000 Split, Croatia; 4 The Doctoral Program in Biophysics, Faculty of Science, University of Split, Ruđera Boškovića 33, 21000 Split, Croatia; ana.bedalov@karmenscience.ai; 5 Bedalov d.o.o for Research, Development and Consulting, Ulica T. Antunovića 17, 21212 Kaštel Sućurac, Croatia

**Keywords:** neuroelectronic interface, microelectrode array, neuronal contact guidance, substrate topography, in vitro, spiral ganglion neurons, spontaneous action potentials

## Abstract

In this study, we present a proof-of-concept neuroelectronic interface (NEI) for extracellular stimulation and recording of neurophysiological activity in spiral ganglion neurons (SGNs) cultured in vitro on three-dimensional, micro-patterned substrates with customized microtopographies, integrated within a 196-channel microelectrode array (MEA). This approach enables mechanotaxis-driven neuronal contact guidance, promoting SGN growth and development, which is highly sensitive to artificial in vitro environments. The microtopography geometry was optimized based on our previous studies to enhance SGN alignment and neuron-electrode interactions. The NEI was validated using SGNs dissociated from rat pups in the prehearing period and cultured for seven days in vitro (DIV). We observed viable and proliferative cellular cultures with robust neurophysiological responses in the form of local field potentials (LFPs) resembling action potentials (APs), elicited both spontaneously and through electrical stimulation. These findings provide deeper insights into SGN behavior and neuron-microenvironment interactions, laying the groundwork for further advancements in neuroelectronic systems.

## 1. Introduction

Microelectrode arrays (MEAs) have been widely used for studying the extracellular electrophysiological properties of neurons in vitro for decades [1,2]. However, their potential for various research applications has only recently gained broader attention [3]. These systems enable simultaneous stimulation and recording from multiple neurons, offering detailed insights into neuronal behavior and fundamental electrophysiological mechanisms. While much of this research has focused on brain-derived neurons [4,5], particularly cortical neurons [6,7], studies on spiral ganglion neurons (SGNs)—the primary auditory neurons in the auditory periphery—remain limited [8,9]. This gap, also induced by a limited number of experimental platforms for in vitro SGN growth and development, hinders our understanding of SGN-electrode interactions, which are critical for addressing key challenges in auditory prosthetics, particularly in cochlear implants (CIs).

Since the earliest recorded cases and studies of mechanotaxis [10,11], contact guidance (CG) between substrates and cellular cultures has aroused interest in testing cell control possibilities under the influence of topographic substrate factors [12]. Advancements in material science in the following decades enabled the development of microstructured substrates that influenced the morphology and behavior of cell cultures imitating the extracellular matrix (ECM) [13], creating extracellular micro and nanoenvironments using specially adapted surface topography to control cellular functions [14] and assessing cell adhesion on micro and nano levels [15,16]. Many studies have investigated the effects of neuronal contact guidance on the neuron responsiveness to mechanical cues of the topography, such as guiding neurite outgrowth, enhancing elongation, and changing morphological properties [17,18], using topography design with grooves and ridges [19,20,21], holes [22] and pillars [23,24,25,26]. The influence of the topography on SGNs was also demonstrated with grooves and ridges on the methacrylate surface [27,28,29,30,31] and with micro-textured nanocrystalline diamond (NCD) pillars [32].

In previous studies, we demonstrated that silicon substrates with micropillars can influence the growth, development, alignment, and morphological characteristics of SGNs compared to flat surfaces [33,34,35]. These studies imply the potential of micropillar-based substrates to promote neuronal elongation and alignment, leading to the development of bipolar morphologies in SGNs.

In this study, the recording of spontaneous activities is included, as spontaneous firing is commonly observed in developing neuronal cultures [36,37]. Electrophysiological spontaneous activity in rodents during the prehearing period has been attributed to the periodic release of ATP from the inner supporting cells, which depolarizes inner hair cells (IHCs), causing the release of glutamate from IHCs onto the afferent dendrites of SGNs and initiating bursts of action potentials (APs) [38,39,40]. While most studies on spontaneous SGN activity in vitro were based on intracellular recording techniques [38,41], only a few studies focused on extracellular recordings using MEA systems [8,9]. These studies, however, contained a planar surface as a substrate for neuron culturing and utilized no microstructured topography.

The ability to differentiate neurites into axons has been demonstrated in hippocampal neurons [42]. In vivo, retinal ganglion cell axons grow directly from polarized cells in the absence of other neurites, while in vitro, in the presence of other neurites, one neurite differentiates into an axon [43]. This process is attributed to the influence of the surrounding environment [44], and further research is needed to better understand this mechanism, particularly in the context of SGNs, which tend to develop bipolar morphologies [45]. The effect of surrounding structures that form scaffolds in the developing mouse cochlea in vivo [46], as well as the use of 3D Matrigel structures combined with neurotrophic factors in SGN cultures in vitro [47], further support the idea that environmental factors play an important role in neuronal polarization.

To our knowledge, this study presents the first system that integrates MEA technology with substrates based on neuronal contact guidance principles, specifically applied to SGNs in the prehearing period, when spontaneous activity is present. By combining these technologies, we aim to bridge the gap between the use of microtopographies to influence neuronal behavior and the ability to record and stimulate electrophysiological activity in vitro. This proof-of-concept system enables the simultaneous recording and stimulation of SGNs, providing valuable insights into their interactions with the microenvironment during early development, and laying the foundation for advancing neuroprosthetic technologies.

## 2. Materials and Methods

**Figure 1:**
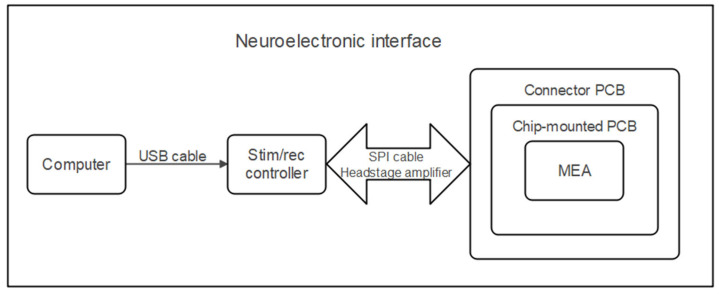
Schematic diagram of the NEI.

The design of the NEI was developed based on our previous morphological studies of in vitro-cultured SGNs, which determined the optimal surface geometry, including the width and spacing of micropillars [33,34,35]. Figure 1 illustrates the schematic of the complete NEI system.

**Figure 2:**
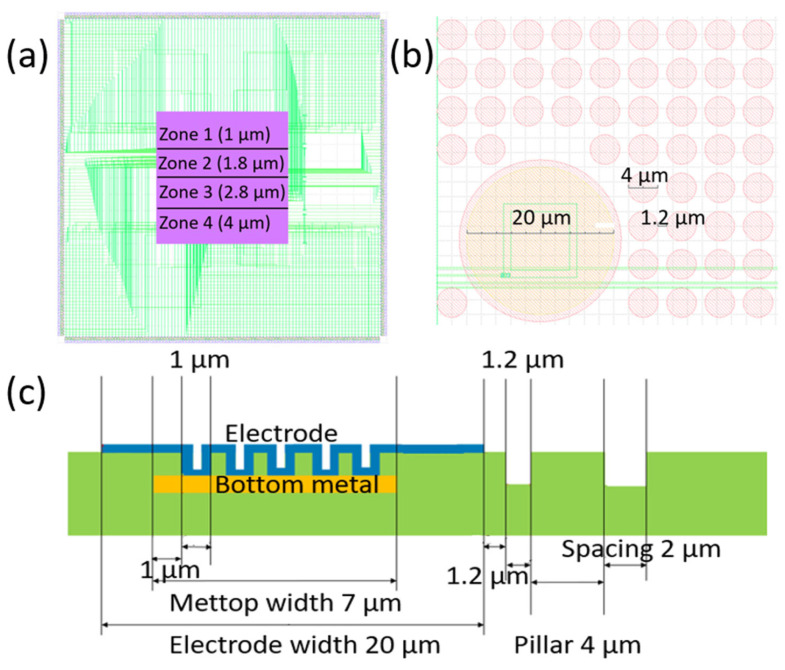
Schematic view of the chip. (**a**) Schematic representation of the pillar zones and connections (depicted as green lines) between the outer pads and the electrodes. The inner square, bounded by the magenta box of 4 µm × 4 µm, represents the active area of the chip containing 4 pillar zones with electrodes. The outer square, bounded by numerous pads shown in gray, is 11 µm × 11 µm in size; (**b**) Top view of a section of the active area in Zone 4, illustrating one electrode (large circle in the bottom left) surrounded by multiple pillars (small circles). Pillar width, spacing, and electrode size are indicated by superimposed numbers in micrometers; (**c**) Side cross-section of a portion of the active area of the chip surface. The base of the chip is a silicon wafer with aluminum conductors 800 nm high (shown in yellow in Panel C and depicted as green lines in Panels A and B) on a 5 µm high SiO_2_ layer (shown in green) on which a 50 nm thick layer of SiC is applied, which serves as a barrier for the etching process. A 3 µm thick SiO_2_ layer (shown in green) was additionally applied to this layer using the high-density plasma chemical vapor deposition (HDPCVD) method. The pillars are shaped by the I-line lithography process. Deep-reactive ion etching (DRIE) method was used to create openings in SiC to obtain an open contact with aluminum. The openings thus obtained are filled with tungsten, and the formation of the electrode is completed by coating a 100 nm thick layer of TiN on top (shown in blue).

The core of the NEI consists of micropatterned chip-like substrates referred to as “chips”. Each chip contains 384 TiN-based electrodes, distributed across 4 distinct pillar zones, each featuring unique geometries and comprising 96 electrodes (Figure 2a). The circle-shaped electrodes are arranged in 4 rows in each pillar zone, with 24 electrodes in each row. The chips were fabricated in collaboration with Imec, Leuven, Belgium.

Due to the limitation in wire bonding of the pads of the chip-mounted PCBs with the chip connection pads, we wire bonded every second electrode row with the chip pads, halving the total NEI electrode capacity to 196 electrodes (i.e., 48 electrodes in each pillar zone). The electrodes are 20 µm wide and spaced 100 µm apart, both within and across rows. Spacing between two adjacent rows is also 100 µm. Each pillar zone contains circle-shaped pillars of different widths (diameters): 1.0 µm (Zone 1), 1.8 µm (Zone 2), 2.8 µm (Zone 3), and 4.0 µm (Zone 4) with the spatial arrangement shown in Figure 2a. Pillars can be spaced apart at two spacing widths: 1.2 µm and 2.0 µm. Pillar spacing is consistent across all areas of the same chip. The height of all pillars is 1 µm. The schematic view of the chip, along with the dimensions of Zone 4, is shown in Figure 2b The cross-section of the chip, showing the electrode geometrical structure, is given in Figure 2c.

**Figure 3:**
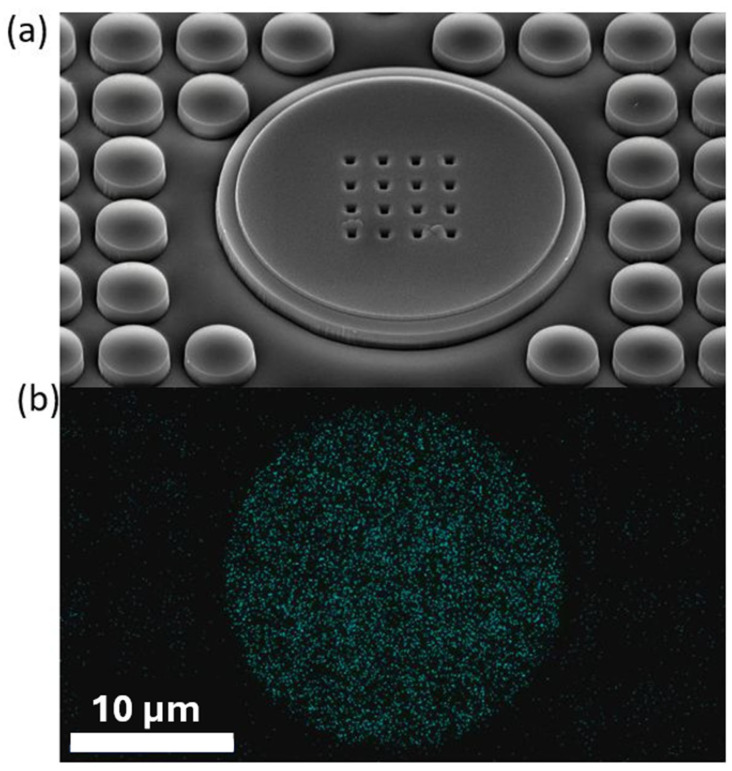
Structure and composition of an active part of the NEI chip. (**a**) SEM image of the chip surface (Zone 1), captured at an angle of 35° with 3000× magnification; (**b**) EDS image of the titanium electrode, where blue dots indicate areas with the strongest signal, confirming the presence of titanium within the TiO_2_ matrix. The scale bar applies to Panel A as well.

Further, we used scanning electron microscopy (SEM) and energy dispersive spectroscopy (EDS) to visualize and assess the composition of the chip (Figure 3). The chip surface was previously coated with a 1-nm-thick layer of platinum with Q 150T ES (Quorum Technologies, Lewes, UK) sputter coater. SEM images were taken with JSM-7610F (JEOL, Tokyo, Japan) at an angle of 35°, and the EDS was carried out perpendicularly with JED-2300 (JEOL, Tokyo, Japan).

The chip is connected to a specifically designed PCB, referred to as a chip-mounted PCB, via a manual wire bonding procedure that employs golden wire (φ 25 µm). This process involves bonding the wire from the PCB pads (70 × 180 µm, spacing: 30 µm) onto the pads located on each side of the chip, utilizing the wire bonder HB05 (TPT, Karlsfeld, Germany). We adhered the chip to the board using a two-component plastic-bond adhesive (Weicon, Münster, Germany), and the wire bonds were subsequently protected with epoxy resin 353 ND (Epoxy Technology, Billerica, MA, USA). A glass ring (φ 40 mm) was affixed to the PCB using silicone adhesive, ensuring the chip was centered to function as a Petri dish for cell cultures.

**Figure 4:**
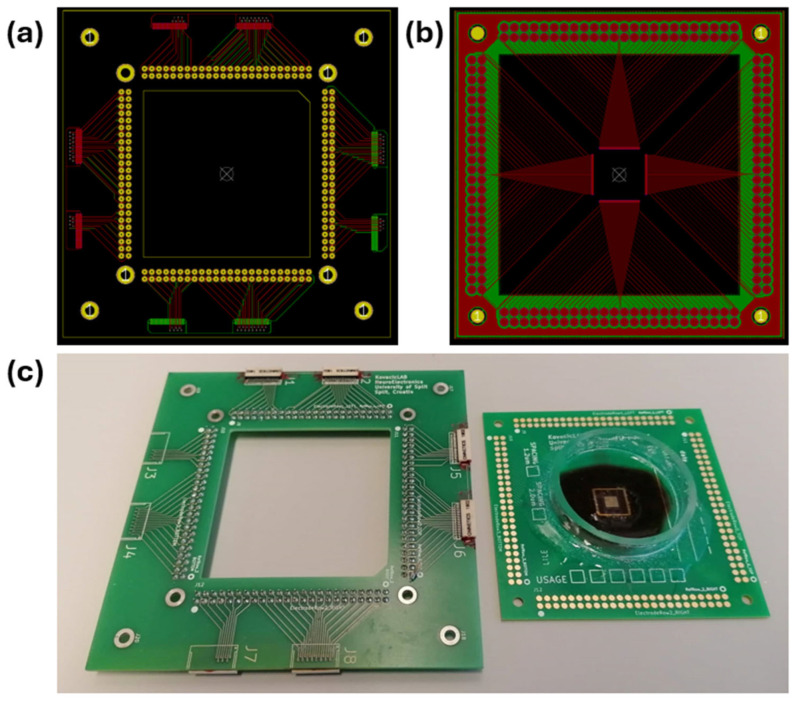
PCB Schematics. (**a**) Connector PCB connecting the stim/rec controller with the chip-mounted PCB. (**b**) Chip-mounted PCB. (**c**) Connector PCB (**left**) and chip-mounted PCB (**right**).

The platform, which supports both the chip-mounted PCB and the connector PCB, is constructed from a steel plate (120 × 120 mm, 2 mm thick). The PCBs are organized in a two-level architecture as presented in Figure 4. The first level accommodates a chip-mounted PCB (80 × 80 mm) that is mounted onto the steel plate. The second level features the connector PCB (110 × 110 mm), which has a central square cutout (55 × 55 mm) and contains two Nano-Miniature Connector adapters (NPD-36-AA-GS from Omnetics Connector Corp., Minneapolis, MN, USA) on each side of the PCB, resulting in a total of eight connectors per PCB. Both PCBs are securely coupled using four M4 nuts and screws. Electrical connections between the PCBs are facilitated by vertically oriented spring pins. Both PCBs were designed using the KiCad PCB design software (version 5.0.2) (KiCad Development Team, Open Source) and fabricated by commercially available PCB manufacturers (Eurocircuits N.V., Mechelen, Belgium, and Ever Star Electronics Pte., Singapore).

We deployed the Intan Stimulation/Recording system RHS 2000 (Intan Technologies, Los Angeles, CA, USA) to control the NEI. The system utilizes a series of miniaturized electrophysiology stimulator-amplifier chips, with the ability to simultaneously record electrophysiological signals and generate independent constant-current stimulation pulses on any or even all channels. The system uses a standard USB cable to connect to a host computer.

The current stimulator works in the current clamp mode. It keeps the current constant while monitoring the changes in voltage. Monophasic, biphasic, and triphasic pulses from ±10 nA up to ±2.55 mA are available for stimulation at sampling rates up to 30 k Samples/s per channel. It is also possible to set the duration of each pulse with a step resolution equal to a time interval between two samples (33.3 µs for 30 kS/s). Importantly, the system provides the automatic limitation of maximal charge injection per pulse and charge-balancing procedure for each triggered pulse to avoid long-term effect charge accumulation. The controller contains four main ports providing access to four headstages being connected via serial peripheral interface (SPI) 16-pin cables. Each headstage contains one 32-channel RHS2116 amplifier chip, connected with a Stim SPI interface cable (diameter 3.4 mm) with a 16-pin Omnetics polarized nano connector.

Electric stimulation delivered to an electrode induces artifacts on neighboring microelectrodes, caused by the capacitive coupling within the MEA, leading to potential electrical crosstalk between adjacent pins (with a capacitance of 0.15 pF between adjacent pins). To prevent crosstalk, we activated the built-in amplifier settling feature across all headstage channels during stimulation. This feature temporarily lowers the bandwidth of the amplifier in order to reduce its sensitivity to higher-frequency components of the artifacts for a brief period before and after the stimulation pulse. A lower bandwidth of 1000 Kz was engaged for 1 ms, following the recommendations of the manufacturer. Random variations in the RHS2116 transistors can disturb charge balance, which may negatively affect cells during long-term chronic experiments. To mitigate the effects of residual charge, we performed charge recovery by grounding each electrode or applying a fixed voltage individually for each electrode/channel. A lower bandwidth frequency of 750 Hz and an upper bandwidth frequency of 7500 Hz were used for signal filtering in order to reduce the reading of lower frequency components in the local field potential (LFP) while simultaneously preserving spikes of APs and reducing noise. The values were determined empirically. Measurements of impedance can be conducted for all electrodes across a wide range of frequencies.

Dissection and culturing of SGNs were performed according to protocols from [48,49] on 6- to 8-day-old (P6–P8) Sprague-Dawley rat pups. After anesthesia with ice and decapitation, a dissecting buffer consisting of phosphate-buffered saline (PBS) with 0.6% glucose (Sigma-Aldrich, St. Louis, MO, USA) and 0.3% bovine serum albumin (BSA; Sigma-Aldrich, USA) was used [50]. Under a microscope, the brain was removed following a mid-sagittal opening of the skull. The temporal bone was then transferred to a dissecting buffer, and the cochlea was isolated after dissection of the otic capsule. The modiolar cartilage and the organ of Corti were then carefully removed.

The enzymatic dissociation of SGNs was performed using 0.25% trypsin-EDTA (Sigma-Aldrich, USA) for 25 min at 37 °C and was stopped by adding DMEM:F12 (Gibco, Thermo Fisher Scientific, Waltham, MA, USA) supplemented with 10% fetal bovine serum (FBS, Biochem, Karlsruhe, Germany) and 38 U/mL DNase (Sigma-Aldrich, USA) in an equal volume. After a 10-min centrifugation at 2000 rpm, trituration was conducted, and the cells were resuspended in a culture medium composed of Neurobasal-A (Gibco, Thermo Fisher Scientific, USA), 1% Pen-Strep (Lonza, Basel, Switzerland), 0.5 mM L-glutamine (Gibco, Thermo Fisher Scientific, USA), B27 supplement (Gibco, Thermo Fisher Scientific, USA), and 30 ng/mL GDNF (Merck Millipore, Darmstadt, Germany) to promote SGN neurite outgrowth [51]. A Bürker-Türk counting chamber was used for cell counting. The cells were seeded at a density of 20,000 cells per sample in a 100 μL volume and allowed to settle for 1–2 h in an incubator (37 °C, 5% CO_2_, 85% humidity). Every 2–3 days, a portion of the medium (50%) was replaced.

Samples were fixed with 4% paraformaldehyde (Thermo Fisher Scientific, Waltham, MA, USA) for 30 min and washed three times with 1X PBS after six to seven days in vitro (DIV). After being washed three additional times with 1X PBS, the culture was prepared for immunostaining and permeabilized with 0.1% Triton X-100 (Millipore, Merck, Germany) for 5 min. The samples were then incubated at room temperature for 90 min in PBS containing 1% normal goat serum (Dako, Santa Clara, CA, USA). Immunostaining was conducted using 1:500 mouse monoclonal anti-βIII tubulin antibody (Millipore, Merck, Germany) for neurons (labeled as Tuj) and 1:500 rabbit polyclonal anti-S100 antibody (Sigma-Aldrich, USA) for glial cells (labeled as S100+). The primary antibodies were incubated overnight at 4 °C. The next day, the samples were washed three times with PBS, followed by a 90-min incubation with secondary antibodies: Alexa 488 goat anti-mouse and Alexa 568 goat anti-rabbit (Thermo Fisher Scientific, Waltham, MA, USA), each diluted 1:500 in 1X PBS with 1% normal goat serum. Nuclear staining was performed with 5 μg/mL DAPI (Molecular Probes, Eugene, OR, USA, Thermo Fisher Scientific, USA) at a 1:500 dilution for 5 min, after which the samples were washed five times with 1X PBS and prepared for imaging using Immu-Mount (Thermo Scientific, Waltham, MA, USA).

**Figure 5:**
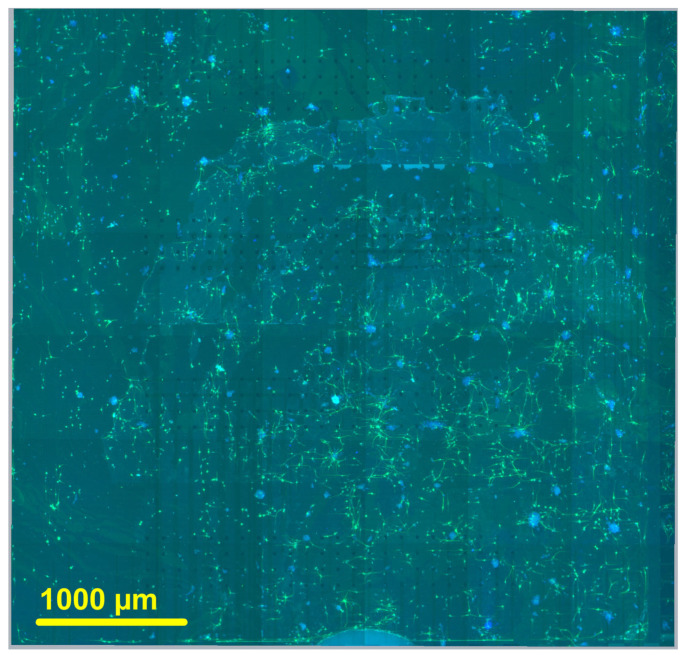
The whole-sample view of the complete MEA surface. It is reconstructed as a 6 × 6 grid map from the individual fluorescence microscopy images of immunocytochemically stained cell cultures grown on the chip. The field of view of each image is taken at 40× magnification. Spiral ganglion neurons were isolated from the P6 rat pups and grown for 7 DIV. The neuronal marker was β-tubulin III, while blue indicated the DAPI marker for cell bodies. The scale bar is shown on the bottom left.

A fluorescent microscope BX61 (Olympus, Tokyo, Japan), paired with a monochromatic CCD Retiga R6 camera (Teledyne, Thousand Oaks, CA, USA), was used for imaging. Sample positioning was controlled with a motorized scanning stage from Prior Scientific (Prior Scientific, Cambridge, UK), operated by software written in µManager (version 2.0.2) (Vale Lab, UCSF, San Francisco, CA, USA). Machine learning-based KARMENscience image analysis platform (Bedalov d.o.o., Kaštel Sućurac, Croatia) was used to overlap images and map them into a single composite image. The chip surface (186 electrodes; areas left and right) with 7 DIV rat pup SGNs stained with Tuj-1 is shown in Figure 5.

The custom-made application programmed in MATLAB (version R2018b) (MathWorks, Inc., Natick, MA, USA) was used to review and analyze the recorded data. The signal width calculated at half the amplitude of the signal distributed over the histogram is used to partially distinguish the spontaneous action potentials (SAPs) from the other components of the LFP. The amplitude distribution for both positive and negative peaks is used to construct profiles of neurophysiological activity providing the differentiation between various signal sources. Interspike interval (ISI) histogram defines a firing pattern of SGNs and can separate different sources of activity [52], both among distinct neurons and between the SAP and other activities. All aforementioned features can be configured with adequate peak amplitude values, peak prominences, polarity, and peak-to-peak distance. Signal similarity is observed through correlation derived from the normalization and comparison of signals. The correlation coefficient between signals from adjacent electrodes can be used to determine whether the signal originates from a common source or several mutually independent activities. The application module for chip analysis enables the visualization of neurophysiological activity across chip electrodes, along with stained neurons grown on the chip at a selected time interval.

## 3. Results

### 3.1. Impedance and Stimulation Artifact Measurement

**Figure 6:**
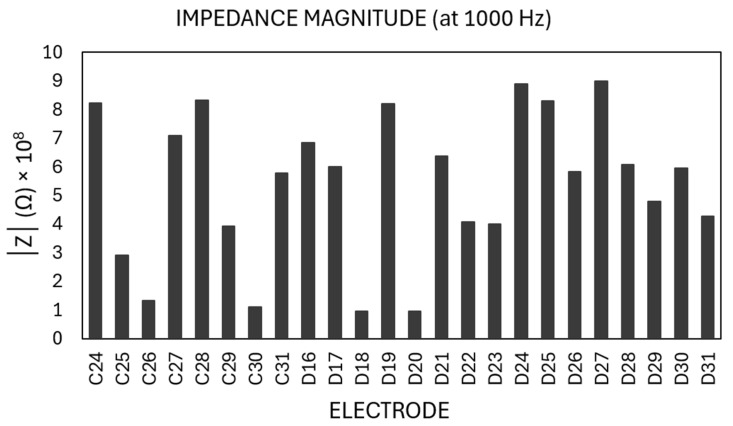
Impedance magnitudes measured at 1 kHz (electrode spacing: 100 µm).

The impedance of the MEA, filled with neurobasal medium, which represents a physiologically similar environment as to that of the medium used for cell cultures, was measured at 1 kHz every 10 s for a total of 10 times to monitor the dynamic change of impedance across the electrodes over time. Representative spatial impedance measurement for one row of electrodes in zone 4 is shown in Figure 6 (mean: 5.37 × 10^8^ Ω, standard deviation: 2.53 × 10^8^ Ω, standard error: 0.52 × 10^8^ Ω, CV: 0.47).

**Figure 7:**
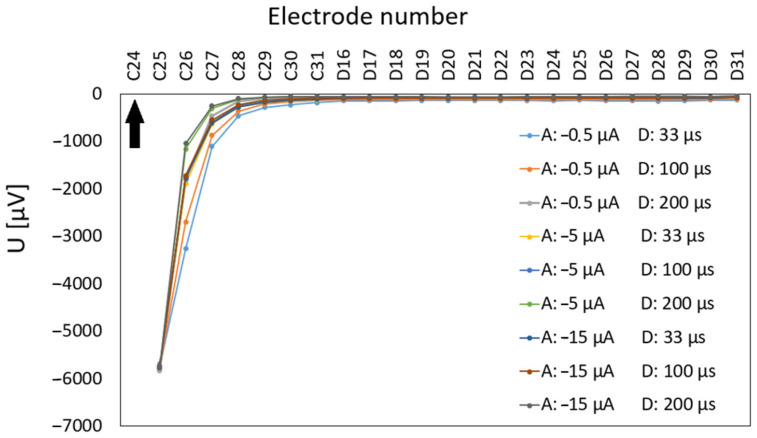
Artifacts of monophasic cathodic stimulation pulse sent to electrode C24 (marked with a black arrow) recorded across the entire electrode row (electrode spacing: 100 µm).

Stimulation artifact measurements on an empty chip with the neurobasal medium were conducted for monophasic cathodic polarity configuration with a stimulation protocol as follows: the pulses were delivered 10 times every 10 s at three amplitude levels (0.5, 5, and 15 µA) and with three pulse durations (33, 100, and 200 µs). Stimulation was performed on the most left electrode of the lower row of zone 2 (electrode C24) and the artifact value was recorded for all 24 electrodes in a row to characterize the system’s ability to read the response signals of the neurons after the stimulation pulse. The highest read values were recorded on the first adjacent electrode of the stimulating electrode (C25) at a distance of 100 µm from the stimulation source and reached values approximately the saturation voltage of the system reading (mean: −5759.90 µV, standard deviation: 30.76 µV, standard error: 3.42 µV, saturation amplitude: ±6389.57 µV). Overall, Figure 7 shows amplitudes decreasing proportionally with the square of the distance, following Coulomb’s law. For example, readings of electrode C29, which is at a distance of 500 µm from the source of stimulation (electrode C24), were significantly lower, approximately following the inverse square law (mean amplitude: −153.50 µV, standard deviation: 66.13 µV, standard error: 7.35 µV). Considering that the readings in extracellular recording techniques are achieved at distances of up to 140 µm [53] (results for hippocampal CA1 pyramidal cells) and the distance between the electrodes is 100 µm, it is to be expected that the majority of recorded action potentials could be recorded within a radius of several electrodes from the source neuron.

### 3.2. Electrical Stimulation and Successful Recording of Local Field Potentials

Electrical stimulation of SGNs was performed at 7 DIV for 1 h after removal from the incubator, for 15 min for each zone on the chip. The order of stimulated zones was randomized for each sample to avoid possible systematic effects of the duration of exposure to conditions outside the incubator. Stimulation was performed with only one electrode in each case to avoid electrical crosstalk and reduce artifacts. Each recording covered an area with 47 recording electrodes and one reference electrode. An aluminum box that also served as a Faraday cage was used as grounding. Recordings were automatically saved in .rhs format in 60-s files to reduce the workload in post-processing. All recordings were merged into larger files if necessary for better clarity.

**Figure 8:**
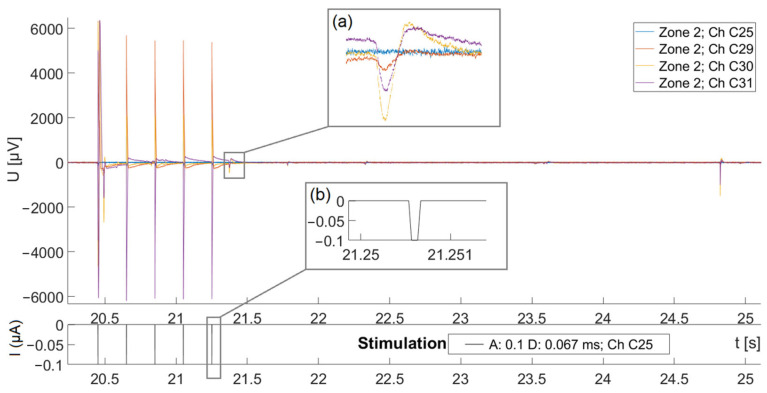
Recording of local field potentials to a train of five monophasic cathodic pulses of amplitude −0.1 µA and phase duration of 67 µs, shown on the bottom, with the inset (**a**) showing the enlarged view of one pulse. Stimulation is delivered on channel C25. The electrical artifacts are clearly seen after each stimulation pulse. The local field potentials are seen approximately 100 ms after the last pulse in the train, with the inset (**b**) showing the enlarged view.

The measured electric response of a single neuron in zone 2 to a monophasic cathodic-first pulse with a phase duration of 67 µs and amplitude of −0.1 µA is demonstrated in Figure 7. Stimulation parameters were determined empirically, whereby in preliminary tests, we tested a range of amplitudes and pulse durations and selected those settings that consistently evoked robust electrophysiological responses in SGN cultures. The electric response of the SGNs on adjacent electrodes after the artifact appears in the form of local field potential, with spikes of the negative polarity, following temporal dynamics typical for neuron’s action potentials. As shown in Figure 8, we observed LFPs, recorded simultaneously on electrodes adjacent to the stimulation electrode, indicating the action potentials of nearby individual spiral ganglion neurons. Given the simultaneous nature of the LFPs across adjacent electrodes, various LFP amplitudes indicate that the SGN might be in the vicinity of these electrodes.

### 3.3. Recording of Spontaneous Electrophysiological Activity

**Figure 9:**
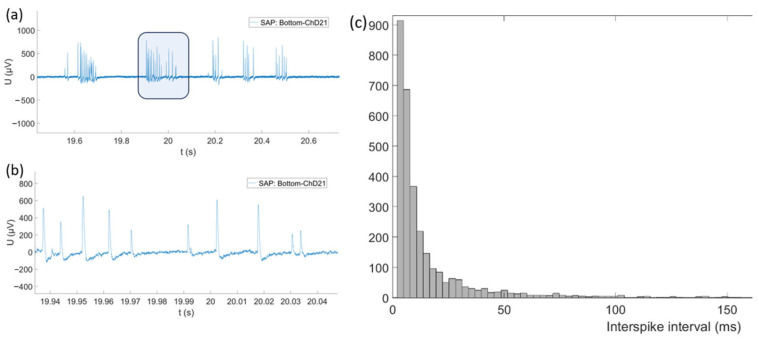
(**a**) View of SAP bursts on a single electrode. (**b**) Zoomed view of a single SAP burst on a single electrode. (**c**) ISI histogram of SAP.

In our pilot experiments to demonstrate the neural activity from primary auditory neurons, we first allowed SGN cell cultures to grow on top of the chip for 7 DIV following our standard in vitro cell culture protocol. After removal from the incubator, the 15-min recordings were made for each zone on the chip with the same recording protocol across four zones. We observed neurophysiological activity in the form of SAPs that mostly occurred in bursts of about 10 to 20 signals per burst. Recorded signals had only positive polarity, as shown in Figure 9a,b. They were observed in zone 4 (N_PEAKS_ = 3100, mean amplitude = 367 µV, standard deviation = 394 µV, above noise threshold = 50 µV). The average signal width at half height was 0.66 ms with a standard deviation of 0.48 ms. The relatively low noise of the system and the highly discernible amplitudes allowed precise detection of spontaneous action potentials from the recorded signals. The histogram of the interspike interval (ISI) obtained from the same recordings in zone 4 (N_PEAKS_ = 3100) is shown in Figure 9c.

**Figure 10:**
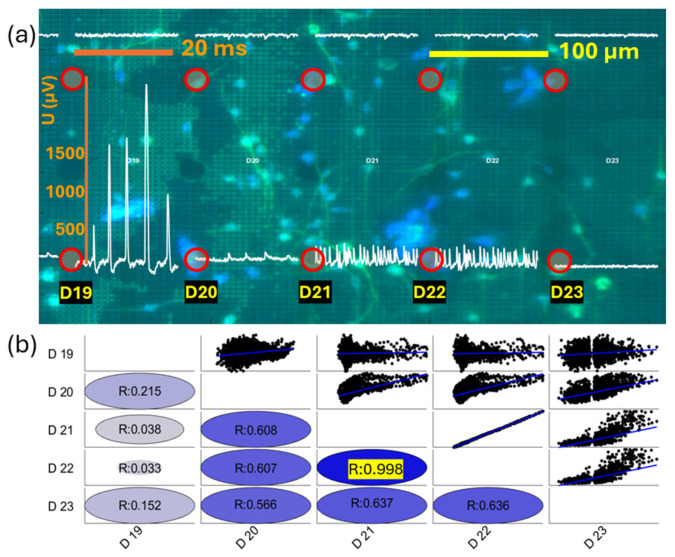
(**a**) Zoomed view of electrophysiological signals superimposed over adjacent electrodes on the neuron-covered MEA surface. SGNs were obtained from P6 rat pups grown for 7 DIV. SGNs are immunocytochemically marked with β-tubulin III, shown in green. Blue represents DAPI-marked cell bodies, which may also belong to glial cells. The field of view was taken at a magnification of 40×. The length scale bar is shown in yellow, and the time scale bar is shown in orange. Red circles represent circle-shaped electrodes at their exact locations. The five electrodes are labeled in black squares (D19, D20, D21, D22, and D23) in the bottom row of the zone. The electrophysiology signals for each electrode are zero-referenced at the electrode location, with the beginning of the signal positioned at the center of the electrode. (**b**) Correlation analyses between signals of the pair of electrodes. The blue ellipses show the correlation strength values of the signals at the electrodes. The black dots represent individual data points from the signals recorded at the paired electrodes, showing the similarity between their amplitude values. The blue line represents the regression line, showing a linear relationship between the values from the corresponding electrode pair.

Next, we present a case of evidence for spatially related spontaneous activity presumably originating from multiple sources and simultaneously recorded on adjacent electrodes. Figure 10a shows readings from five adjacent electrodes (designated as D19, D20, D21, D22, and D23) from the same row in zone 4. These readings might be associated with spatially distributed neural activity from multiple sources. The leftmost electrode (D19) displayed LFPs with patterns similar to single-cell action potentials with varying amplitudes. The next electrode (D20), separated by 100 um, displayed a seemingly associated activity, as seen on D19, since spikes appeared simultaneously. In contrast, the following two electrodes that were further in the right direction (D21 and D22) showed entirely different patterns from the first two in terms of amplitude values, number of peaks, and spiking times. Finally, we observed, with the correlation analyses described below, that the electrophysiological signal on the rightmost electrode, D23, largely coincides with the signal from the preceding two electrodes (D21 and D22) but with significantly smaller amplitudes, which is consistent with the electrode being further away from the neuron, presumably generating the signal. To determine whether the signals from different electrodes belong to the same neuron, we performed correlation analyses of the signals from different electrodes. Figure 10b shows correlation coefficients of the signal similarity between the pair of electrodes, shown in circles, together with the point-by-point value comparisons of pairs of electrodes, shown as dotted scatter graphs. The strong correlation (r_D21–D22_ = 0.998), seen between the adjacent electrodes D21 and D22, seems to point to a cluster of neurons visually detected between these two electrodes as a signal source. Compared with the signals from two adjacent electrodes on each side, i.e., the electrodes D20 and D23, we observe a weaker correlation between them (r_D20–D21_ = 0.608, r_D22–D23_ = 0.636). Next, we compared the signals on electrode D19 with the neighboring electrode D20, which brings a moderate correlation (r_D19–D20_ = 0.215), while there is almost no correlation with signals from the electrodes D21 and D22 (r_D19–D21_ = 0.038 and r_D19–D22_ = 0.033). A slightly stronger correlation occurs with the more distant electrode D23, separated by 400 µm (r_D19–D23_ = 0.152), which suggests that signals of a particularly strongly active neuron may be registered with quite distant electrodes. Yet, on closer electrodes, such as D21 and D22, its presence is presumably masked by the influence of nearby local sources, closer to the D21 and D22. Overall, these comparisons provide the existence of multiple independent sources, putatively belonging to different SGNs, showing spontaneous neurophysiological activity on different electrodes simultaneously.

## 4. Discussion

Severe hearing loss with sensorineural etiology affects approximately 430 million people worldwide requiring auditory rehabilitation [54], posing significant social and economic challenges. CIs have proven to be one of the most effective solutions, with more than one million units implanted globally by 2022 [55]. However, the outcomes of implantation are still highly variable and often unpredictable [56]. One of the main reasons for CIs’ suboptimal performance is the lack of precise stimulation caused by the neuroanatomical gap (NAG); the distance between the electrodes and SGNs in the cochlea [57,58], which is further emphasized considering that the human cochlea contains typically about 30,000 SGNs [59], while most modern implants do not exceed 24 electrodes [56]. These spatial and numerical discrepancies can severely reduce the electrical coupling (EC) efficiency, causing poor pitch perception and speech perception in noise [60,61], since the effect of stimulation heavily relies on EC, which depends on the position of the electrode and the distance between electrodes and neurons [56,62]. While reducing the NAG is a long-term goal [63], foundational studies are needed to address challenges such as limited spatial resolution in stimulation delivery difficulties in precisely confining electrical fields emphasizing the need for improved platforms to understand better neuron-electrode interactions at both the cellular and network levels [64].

## 5. Conclusions

In this study, we presented the first NEI for extracellular stimulation and high-resolution recording of multiple independent signal sources of SGNs’ activity, with micropillar-based microtopography optimized to support SGN growth and guide neurite extension through mechanotaxis in a way that could reduce the spatial discrepancies between neurons and electrodes, potentially improving EC and thus reducing energy expenditure of the CI with comparable number of stimulation electrodes. This feature represents a step forward over existing systems for analyzing the electrophysiology of SGNs extracellularly. SGNs exhibited spontaneous neurophysiological activity characteristic of their developmental stage and responsiveness to electrical stimulation, confirming the suitability of NEI for studying neuron-electrode interactions. The results highlight the potential of substrates with micro-patterned topography in shaping neuronal behavior in vitro.

For continued research and potential applications, the long-term stability and biocompatibility of the substrate material and its ability to maintain the quality of recordings and stimulation of neurons will be of great importance. Future studies will also need to examine the survival rate and electrophysiological behavior of neuronal cultures over longer periods, for example at DIV 14 or DIV 21. The uniformity of impedances on the electrodes, a parameter essential for the reliability of the readings, will depend on the quality and stability of the manufacturing process. The high scalability of the production process enables the rapid production of a large number of samples and, with design modifications, changes in chip dimensions. A higher electrode density would contribute to a higher resolution of electrophysiological recordings on the sample, which would allow for more reliable activity analyses, and a detailed analysis of the stimulation threshold would contribute to the optimization of the stimulation protocol and the efficiency of the system. A comparison of the properties of electrical activity on a flat MEA substrate in relation to a substrate with micropillars would expand the understanding of the direct influence of microstructures on the electrophysiological properties of neurons. The performance of the electrodes could be improved by improving the chip manufacturing process and standardizing the process of connecting NEI components, especially solder pins on PCBs and wire bonding. The spontaneous activity of dissociated SGNs in the absence of IHCs opens new questions about their intrinsic electrophysiological properties. These findings could contribute to the broader understanding of neuron-electrode interactions and pave the way for the development of advanced MEA-based neuroelectronic interfaces.

## Data Availability

The data supporting the findings of this study, including neurophysiological recordings, are available from the corresponding author upon reasonable request.

## Abbreviations

The following abbreviations are used in this manuscript:

MEA	Microelectrode array
SGN	Spiral ganglion neuron
CG	Contact guidance
CI	Cochlear implant
ECM	Extracellular matrix
NCD	Nanocrystalline diamond
IHC	Inner hair cell
AP	Action potential
NEI	Neuroelectronic interface
PCB	Printed circuit board
SEM	Scanning electron microscopy
EDS	Energy dispersive spectroscopy
SPI	Serial peripheral interface
LFP	Local field potential
PBS	Phosphate-buffered saline
BSA	Bovine serum albumin
DIV	Days in vitro
SAP	Spontaneous action potential
ISI	Interspike interval
NAG	Neuroanatomical gap
EC	Electrical coupling
